# Symptoms of Eating Disorders and Depression in Emerging Adults with Early-Onset, Long-Duration Type 1 Diabetes and Their Association with Metabolic Control

**DOI:** 10.1371/journal.pone.0131027

**Published:** 2015-06-29

**Authors:** Christina Bächle, Karin Lange, Anna Stahl-Pehe, Katty Castillo, Nicole Scheuing, Reinhard W. Holl, Guido Giani, Joachim Rosenbauer

**Affiliations:** 1 German Diabetes Center, Institute for Biometrics and Epidemiology, Düsseldorf, Germany; 2 German Center for Diabetes Research (DZD), Neuherberg, Germany; 3 Hannover Medical School, Department of Medical Psychology, Hannover, Germany; 4 University of Ulm, Institute of Epidemiology and Medical Biometry, ZIBMT, Ulm, Germany; Baylor College of Medicine, UNITED STATES

## Abstract

**Background:**

This study analyzed the prevalence of and association between symptoms of eating disorders and depression in female and male emerging adults with early-onset, long-duration type 1 diabetes and investigated how these symptoms are associated with metabolic control.

**Methods:**

In a nationwide population-based survey, 211 type 1 diabetes patients aged 18-21 years completed standardized questionnaires, including the SCOFF questionnaire for eating disorder symptoms and the Patient Health Questionnaire (PHQ-9) for symptoms of depression and severity of depressive symptoms (PHQ-9 score). Multiple linear and logistic regression models were used to analyze the association between eating disorder and depressive symptoms and their associations with HbA1c.

**Results:**

A total of 30.2% of the women and 9.5% of the men were screening positive for eating disorders. The mean PHQ-9 score (standard deviation) was 5.3 (4.4) among women and 3.9 (3.6) among men. Screening positive for an eating disorder was associated with more severe depressive symptoms among women (β_women_ 3.8, p<0.001). However, neither eating disorder symptoms nor severity of depressive symptoms were associated with HbA1c among women, while HbA1c increased with the severity of depressive symptoms among men (β_men_ 0.14, p=0.006).

**Conclusions:**

Because of the high prevalence of eating disorder and depressive symptoms, their interrelationship, and their associations with metabolic control, particularly among men, regular mental health screening is recommended for young adults with type 1 diabetes.

## Introduction

Everyday challenges for patients with childhood-onset type 1 diabetes mellitus (T1D) include the regular monitoring of blood glucose; the balancing of insulin administration, food intake and physical activity; and the fear of acute and late diabetes-related complications. From an early age, these patients are at a higher risk of depression and eating disorders than their peers without diabetes [1].

The prevalence of depression in patients with T1D is rapidly increasing, and diabetes and depression are predicted to become the most common health problems in the 21^st^ century [2]. However, data regarding the prevalence of depression in people with T1D remain scarce. In a systematic review summarizing the results of studies and review articles published between 2006 and 2011, only two articles focused on the prevalence of depression in people with T1D [3]. One study was a meta-analysis estimating the prevalence of clinical depression among adults with T1D between 2000 and 2004. People with T1D were four times more likely to have clinical depression than people without T1D (pooled prevalence 12.0% vs. 3.2%) [4]. Analyzing studies without a control group, the pooled prevalence was 13.4%. The other study reported that 32.1% of the adults with T1D (women: 37.9%, men: 25.5%) were screening positive for depression (Beck Depression Inventory II) or used antidepressants compared with 16.0% of the adults without T1D (women: 20.5%, men: 11.6%) [5]. In another study using the same screening tool, the prevalence of adolescents and young adults (age 11–25 years) with T1D screening positive for depression was analyzed. Here, 11.3% of the participants screened positive for depression, however, the sample size was small (150 participants) and the age range wide [6].

Many previous studies have shown higher HbA1c levels in T1D patients with depressive symptoms [6–10], whereas a few studies have been inconclusive [11–13] or have found no such association [14,15]. Recently, we reported that, while the prevalence of depressive symptoms was higher among young adult women with T1D than among men, the association between single depressive symptoms and metabolic control was stronger among men [16]. However, gender-specific analyses have been rarely performed. The coexistence of diabetes and depression has been found to increase mortality in T1D women [17] and diabetes-associated complications [9,18,19].

In addition, diabetes therapy and changes in body weight during puberty, which are more pronounced in T1D patients than in their healthy peers [20], increase the risk of persistent eating problems (both disordered eating behavior [DEB] and eating disorders [EDs]) [21]. According to a systematic review with a meta-analysis summarizing the results of eight studies published between 1999 and 2011, both DEB and EDs were more prevalent in children, adolescents and young adults with T1D than in peers (DEB: 39.3% vs. 32.5%, EDs: 7.0% vs. 2.8%) [22]. In Germany, screening prevalences of EDs in 11- to 17-year-old adolescents with early-onset T1D and at least ten years of diabetes duration were 31.2% for girls and 11.7% for boys compared with 28.9% for girls and 15.2% for boys from the general population [23]. Thus, the previously reported female preponderance for ED symptoms in the general population [24,25] was also observed among adolescents with T1D. However, results relating to the prevalence of ED symptoms among male patients with T1D are rare.

ED screening is only the first step in ED diagnosis and should be followed by in-depth examination in clinical practice. The prevalence of a clinical diagnosis of EDs has recently been published based on the data on 52,215 8- to 29-year-old patients with T1D from Germany and Austria [26]. The ED prevalence documented in the participants’ medical data was 0.9% and thus much lower than the screening results. However, because of potential underreporting in the database, the real prevalence of clinically manifest EDs may be higher.

Once established, eating problems are frequently associated with poor metabolic control. Summarizing the results from eleven empirical studies, Young et al. observed a significant association between eating problems and metabolic control (Cohen’s d = 0.40) in adolescents which was more pronounced after restricting the analysis to studies using diabetes-adapted measures (d = 0.52) [22]. Current or previous eating problems in female adolescents and young adults increased the odds (by 4.8 to 9.6) of having at least two serious diabetes complications (laser-treated retinopathy, preproliferative retinopathy, peripheral nephropathy, autonomic neuropathy, proteinuria, or renal failure) at follow-up after 8 to 12 years and have been associated with high mortality [27].

Although symptoms of depression and EDs are common in young patients with T1D, their interrelationship and effect on metabolic control remain largely unexplored [1]. Eating problems may directly or indirectly impair metabolic control by inducing depressive disorders. Only one recent study analyzed the association between depressive symptoms, DEB and metabolic control in teenage girls (baseline age 9–14 years, follow-up after 5 years) [1]. Depressive symptoms and DEB were found frequently in this patient group and often co-occurred. At follow-up, the prevalence of current depressive symptoms was 12.2%. Almost half of the participants (49.0%) screened positive for DEB and 13.3% were categorized as having subthreshold or full EDs. A total of 69.2% of the girls who screened positive for DEB reported depressive symptoms, and 75.0% of the girls with depressive symptoms also screened positive for DEB. Adjusting for the baseline symptoms of depression and EDs, the authors found that diabetes duration and body mass index (BMI), eating status and depression status at follow-up were significantly associated with metabolic outcomes (R^2^ = 0.100, p = 0.01). The association between depressive symptoms, eating problems and metabolic control in male patients and young adults who may be particularly challenged by age-related changes in living conditions has not been examined to date.

Although young people aged 18 and older are considered adults, the mental development of young adults is not complete until many years later in industrialized and post-industrialized countries [28–30]. The developmental stage between 18 and 30 years is often termed “emerging adulthood” and is characterized by frequent changes in residency, exciting experiences, new freedoms and wide-open possibilities as well as uncertainty, setbacks, confusion, and new fears [29]. While young people adopt adult roles and cope with educational, economic and social challenges, they are increasingly responsible for the many facets of appropriate diabetes care [28]. Additionally, the transition to adulthood is associated with a transition from pediatric to adult diabetes care, which introduces challenging diabetes management. As shown in a subsample of 185 participants from the SEARCH for Diabetes in Youth Study, the odds for poor diabetes control (i.e., HbA1c ≥9% [corresponding to ≥75 mmol/mol]) increased 2.46-fold for people who left pediatric diabetes care [31]. Achieving and maintaining good metabolic control at a young age, however, is fundamental for the prevention or delay of the onset of diabetic complications, as demonstrated by the results of the Diabetes Control and Complication Trial (DCCT) and the Epidemiology of Diabetes Interventions and Complications (EDIC) Study [32–37]. Suboptimal metabolic control during adolescence and young adulthood has been attributed to physiological [38], social [4], and individual factors such as poor adherence to treatment and psychological comorbidities (e.g., EDs and depression) [11,39]. Many studies have examined the effects of diabetes on adolescents’ mental health, but few studies have reported on these effects on young adults’ mental health [30].

Although the incidence of early-onset T1D has been increasing in recent decades [40], its physiological and psychological consequences in young female and male adults are largely unknown. There are two contradictory hypotheses: (1) Young adults with early-onset diabetes are better adjusted to the demands of diabetes and thus have fewer mental health problems than patients with later disease onset and briefer duration; and (2) young adults with early-onset diabetes are at an increased risk for diabetes complications [41] and are therefore particularly vulnerable to mental health problems. The postulated vicious circle between psychiatric and diabetes-related health problems [28] and the high persistence of psychiatric problems [14] reveal the relevance of this research issue.

Therefore, the aim of this study was (1) to analyze the prevalence of EDs and (2) its association with depressive symptoms in a well-defined group of young adults with early-onset, intensely treated T1D of a long duration and (3) to estimate the associations between symptoms of EDs, depressive symptoms and metabolic control. We hypothesized that (1) symptoms of EDs and depressive symptoms are more frequent in women with T1D than in men with T1D; (2) ED symptoms are associated with depressive symptoms; and (3) both ED and depressive symptoms are associated with worse metabolic control.

## Materials and Methods

### Study population

The “Clinical Course of Type 1 Diabetes in Children, Adolescents and Young Adults with Disease Onset in Preschool Age” study is a nationwide, population-based cohort study in Germany. The baseline survey was conducted between September 2009 and December 2010 and has been previously described in detail [42]. In brief, all patients with early diabetes onset (ages 0–4 years) during the 1993–1999 period and their legal guardians (in the case of minors) were contacted via their treatment facilities. Patients and parents/guardians were asked to answer extensive standardized questionnaires (for 11- to 17-year-old adolescents and their parents and for 18- to 21-year-old adults) and to return them with their written informed consent. The ethics committee of the Heinrich Heine University Düsseldorf had approved the study in advance.

The current analyses used data from 18- to 21-year-old young adults. Of the 1,212 eligible young adults, 746 (62%) patients were invited by their treatment facilities to participate in the study. Of these patients, 211 young adults (85 men and 126 women) returned the extensive questionnaire, resulting in a 28% response rate.

### Assessment of variables

The brief German version of the Patient Health Questionnaire (PHQ-9) was used to assess depressive symptoms based on DSM-IV and ICD-10 criteria [43]. The PHQ-9 is also consistent with the recently introduced DSM-5 criteria. Therefore, the PHQ-9 was recommended as an emerging measure of depression severity in adults [44].

The PHQ-9 assesses nine core depressive symptoms (anhedonia, depressed mood, sleep difficulty, lethargy, overeating/poor appetite, low self-esteem/feeling of worthlessness, concentration difficulties, psychomotor retardation/agitation, and suicidal ideation) in the preceding two-week period. The four response categories range from “not at all” (0 points) to “nearly every day” (3 points) [45]. Summing up the values of all answers results in an estimated score for severity of depressive symptoms (range 0–27). Scores of 0–4, 5–9, 10–14, 15–19 and ≥20 indicate no, mild, moderate, moderately severe, and severe depressive symptoms, respectively [43].

The German version of the PHQ-9 was developed in collaboration with the authors of the original version and was validated in a sample of medical and psychosomatic outpatients. Here, the PHQ-9 showed the best operating characteristics regarding major depressive disorder (cutoff value ≥11: sensitivity 98%, specificity 80%) and any depressive disorder (cut-off value ≥9: sensitivity 87%, specificity 76%) compared with the Hospital Anxiety and Depression Scale (HADS) and the WHO (five) Well Being Index (WBI-5) [46]. The PHQ-9 has also been validated for outpatients with diabetes in the Netherlands. With the Mini-International Neuropsychiatric Interview (M.I.N.I.) as a reference method for major depressive disorder, the sensitivity and specificity of the PHQ-9 were 91.9% and 64.4% for a cut-off value ≥10 and 75.7% and 80.0% for a cut-off value ≥12, respectively [47].

The SCOFF questionnaire was used to screen for eating problems. Using five questions with dichotomous answers (yes/no), the survey assesses the following main characteristics of EDs: intentional vomiting, loss of control over food, unhealthy weight loss, body image disturbance, and intrusive food thoughts [48,49]. If two or more questions are answered in the affirmative, then an ED is suspected. In validation studies, estimates of the sensitivity and specificity of the SCOFF questionnaire have ranged from 73% to 100% and from 21% to 94%, respectively [50].

All covariates were obtained from the participants’ questionnaire data. The participants were assessed as having a low, middle or high socioeconomic status (SES) according to the results of a composite social status index based on education level, professional status, and household income [51,52]. For participants who were not the principal earner in their households (primarily because they had not completed professional training), the data for the household’s principal earner were used. Information on family structure/residence indicated whether the participants were living with both parents, with one parent (and his/her partner) or in a separate household alone or with a partner. BMI was calculated based on the participants’ self-reported height and weight, and participants were categorized as underweight/normal weight (<25 kg/m^2^), overweight (≥25-<30 kg/m^2^), or obese (≥30 kg/m^2^) according to the WHO recommendation [53]. Furthermore, data on the self-reported most recently measured HbA1c value were included (absolute value and categorized as <7.5%, 7.5%-9.0% and >9.0%, corresponding to <58 mmol/mol, 58–75 mmol/mol and >75 mmol/mol, respectively).

### Statistical analyses

Continuous variables are described as means with standard deviations (SDs), and categorical variables are described as proportions. The distribution of severity of depressive symptoms in female and male patients with and without ED symptoms is presented in a cross-table, including the results of Fisher’s exact test. To adjust for confounders, the associations between ED and depressive symptoms were further examined using multiple linear regression models with the PHQ-9 score as the dependent variable and SCOFF positivity (potential ED) as the independent variable. All models were fitted stratified by gender. First, the crude model (M1) was fitted. Model 2 (M2) was adjusted for age and diabetes duration, and Model 3 (M3) also included BMI, SES and family structure/residence. Furthermore, multiple linear models were used to analyze the association between metabolic control as reflected by HbA1c and symptoms of EDs and depression severity (total PHQ-9 score). Taking HbA1c as the dependent variable, the crude Models 1 and 2 (M1 [SCOFF], M2 [PHQ-9 score]) were first combined (M3), then adjusted for age and diabetes duration (M4), and finally adjusted for BMI, insulin pump therapy, SES and family structure/residence (M5). Results of the regression analyses are presented as regression coefficients with 95% confidence intervals (CIs) and p-values of respective Wald tests. Two-tailed p-values <0.05 were considered statistically significant. The analyses were performed using SAS for Windows Version 9.4 (SAS Institute, Cary, North Carolina, USA).

## Results

### Description of the sample

The mean age of the participants was 19.4 (SD 1.0) years, the mean age of onset was 3.7 (0.9) years, and the mean diabetes duration was 15.7 (1.0) years, with no difference observed between men and women. Compared with the 1,001 non-participants (including 535 invited and 466 unreachable subjects), the proportion of participating men was 13.2% lower (percentage of men: 40.3% vs. 53.5%, p<0.001). Participants were 0.18 years younger (p = 0.017), they had a similar age of onset (0.02 years older, p = 0.789), and their diabetes duration was 0.20 years briefer (p = 0.010).

With an average HbA1c of 8.6 (1.7) % (corresponding to 70 [19] mmol/mol), women tended to have higher HbA1c values than men (8.1 [1.4] %, i.e., 65 [15] mmol/mol, p = 0.061) and a less favorable distribution of the different HbA1c categories (p = 0.037). Further details regarding the sample are presented in Table 1.

**Table 1:** Characteristics of the study population.

	Men^1^	Women^1^	p-value
N	85	126	
Age [years]	19.3 (0.9)	19.4 (1.0)	0.347^2^
Age of diabetes onset [years]	3.6 (1.0)	3.8 (0.9)	0.1052
Diabetes duration [years]	15.7 (1.1)	15.7 (1.0)	0.5642
HbA_1c_ [%]	8.1 (1.4)	8.6 (1.7)	0.0612
[mmol/mol]	65 (15.3)	70 (18.6)	
<7.5% (<58 mmol/mol; optimal)	**40.5**	**23.2**	**0.037** ^**3**^
7.5%-9.0% (58–75 mmol/mol; suboptimal)	**34.2**	**47.3**	
>9.0% (>75 mmol/mol; high risk)	**25.3**	**29.5**	
Insulin therapy regimen [%]			
Conventional therapy (1–3 daily injections)	4.7	8.7	0.436^3^
Multiple daily injections (≥4 daily injections)	50.6	52.4	
Continuous subcutaneous insulin infusion	44.7	38.9	
BMI [kg/m^2^]	**23.0 (3.0)**	**24.1 (3.0)**	**0.006** ^**2**^
Underweight (i.e., BMI<18.5) [%]	2.4	0.8	0.259^3^
Normal weight (i.e., BMI 18.5-<25) [%]	76.5	69.8	
Overweight (i.e., BMI ≥ 25) [%]	21.2	29.4	
Socioeconomic status [%]			
Low	**17.7**	**33.3**	**0.027** ^**3**^
Middle	**48.2**	**43.7**	
High	**34.1**	**23.0**	
Family structure [%]			
Living with both biological parents	**75.3**	**58.7**	**0.013** ^**3**^
Living with mother or father (and her/his partner)	**18.8**	**20.6**	
Living alone/with partner in an apartment	**5.9**	**17.5**	
Other	**0.0**	**3.2**	
Number of screening positive symptoms for ED per patient [%]			
0	**62.4**	**35.7**	**0.001** ^**3**^
1	**28.2**	**34.1**	
2	**8.2**	**23.8**	
3	**1.2**	**4.8**	
4	**0.0**	**1.6**	
5	**0.0**	**0.0**	
Screening results for SCOFF [%]			
ED (screening positive for ≥ 2 items)	**9.5**	**30.2**	**<0.001** ^**3**^
Number of screening positive symptoms for depression per patient [%]			
0	66.7	57.0	0.441^3^
1	14.8	14.1	
2	4.9	12.4	
3	6.2	5.0	
4	4.9	3.3	
5	2.5	5.0	
6	0.0	1.7	
7	0.0	0.0	
8	0.0	1.7	
9	0.0	0.0	
Screening results for PHQ-9 [%]			
At least moderate depression severity	9.9	14.0	0.514
(PHQ score ≥10)			
PHQ-9 Score	**3.9 (3.6)**	**5.3 (4.4)**	**0.018** ^**2**^

^1^ Data are presented as n, mean (SD), and %

^2/3^ p-value of t-test/Fisher’s exact test for comparison of male and female patients

### Screening prevalences of eating problems and depression (hypothesis 1)

The data showed a female preponderance for ED symptoms and higher rates of positive screening for two or more ED symptoms among women than among men (Table 1). However, no difference between women and men was observed for the distribution of the number of positive PHQ-9 items, although the mean PHQ-9 score was slightly higher among women than among men. A total of 14.0% of the women and 9.9% of the men (p = 0.514) screened positive for at least moderate severity of depressive symptoms (i.e., PHQ-9 ≥10)

### Association between ED and severity of depressive symptoms (hypothesis 2)

According to bivariate analyses, 7.9% of women and 2.4% of men were screening positive for both ED and at least moderate severity of depressive symptoms (p = 0.129). There was a tendency for more severe depression among women than among men and greater severity in patients who screened positive for EDs than in patients without ED symptoms (Table 2). Among women who screened positive for an ED, 27.8% had at least moderate severity of depressive symptoms, compared with only 8.2% of the women without ED symptoms of (p = 0.009). The respective prevalences among men were 25.0% and 8.3% (p = 0.181). The mean PHQ-9 score was 7.8 among women with ED symptoms and 4.2 among women without ED symptoms (p<0.001). The figures for men were 5.8 and 3.7 (p = 0.127).

**Table 2:** Severity of depression symptoms among patients screening positive for eating disorders (EDs) and control patients.

	Male patients			Female patients		
	No ED	ED	p-value	No ED	ED	p-value
Severity of depression symptoms						
no	**69.4**	**37.5**	**0.049** ^**1**^	**58.8**	**27.8**	**0.003** ^**1**^
mild	**22.2**	**37.5**		**32.9**	**44.4**	
moderate	**8.3**	**12.5**		**7.1**	**16.7**	
moderately severe	**0.0**	**12.5**		**1.2**	**8.3**	
severe	**0.0**	**0.0**		**0.0**	**2.8**	
PHQ-9 Score	**3.7**	**5.8**	**0.127** ^**2**^	**4.2**	**7.8**	**<0.001** ^**2**^

^1/2^p-value of Fisher’s exact test/ t-test for comparison of ED positive and control patients

In accordance with the results of the multiple regression analysis presented in Table 3, SCOFF positivity was associated with increased PHQ-9 scores (dependent variable) in women (β_SCOFF M3 women_ 3.8, p<0.001). Furthermore, there was a trend for higher PHQ-9 scores among men screening positive for an ED (β_M3 men_ 2.3, p = 0.068). Severity of depressive symptoms was increased in men living with a mother/father (and her/his partner) (β_M3 men_ 2.5, p = 0.014) or alone/with their partners in an apartment (β_M3 men_ 5.2, p = 0.003) when compared with those who lived with both parents. Among women, neither family structure/residence nor any other covariate was associated with severity of depressive symptoms.

**Table 3:** Multiple linear regression models for the association between depression severity (dependent variable) and SCOFF positive screening results for eating disorders.

	Model 1		Model 2		Model 3	
	ß (95% CI)	p	ß (95% CI)	p	ß (95% CI)	p
Male patients						
SCOFF positive	2.056 (-0.594; 4.705)	0.127	2.208 (-0.472; 4.889)	0.105	2.344 (-0.178; 4.866)	0.068
age			0.606 (-0.387; 1.599)	0.228	0.688 (-0.287; 1.663)	0.164
diabetes duration			-0.452 (-1.336; 0.433)	0.312	-0.717 (-1.548; 0.114)	0.090
BMI					-0.123 (-0.385; 0.139)	0.352
socioeconomic status (reference: high)						
low					1.963 (-0.283; 4.209)	0.086
middle					0.445 (-1.814; 2.704)	0.695
family structure/residence: living …						
(reference: with both parents)						
with mother or father (and her/his partner)					**2.483 (0.520; 4.446)**	**0.014**
alone or with partner in an apartment					**5.229 (1.873; 8.584)**	**0.003**
Female patients						
SCOFF positive	**3.606 (2.330; 5.070)**	**<0.001**	**3.644 (2.033; 5.257)**	**<0.001**	**3.785 (2.061; 5.508)**	**<0.001**
age			-0.534 (1.490; 0.422)	0.271	-0.670 (-1.745; 0.404)	0.542
diabetes duration			0.510 (-0.413; 1.433)	0.276	0.622 (-0.331; 1.576)	0.198
BMI					-0.229 (-0.477; 0.019)	0.070
socioeconomic status (reference: high)						
low					0.067 (-1.657; 1.790)	0.939
middle					-1.537 (-3.647; 0.574)	0.152
family structure/residence: living …						
(reference: with both parents)						
with mother or father (and her/his partner)					0.741 (-1.151; 2.633)	0.440
alone or with partner in an apartment					0.811 (-1.528; 3.150)	0.493
other					2.076 (-2.409; 6.562)	0.361

Data are presented as regression coefficient β (95% CI) with p-value. Model 1: SCOFF (ED) (unadjusted). Model 2: Model 1 + age + diabetes duration. Model 3: Model 2 + BMI + socioeconomic status + family structure/residence^#^

### Associations between positive screening results of depression and EDs and metabolic control (hypothesis 3)

Table 4 illustrates the association between screening positivity for EDs, the PHQ-9 score, and HbA1c (dependent variable). Higher PHQ-9 scores were significantly associated with increased HbA1c in men. In the final model (M4), a one-unit increase in the PHQ-9 score increased the mean HbA1c by 0.14% (1.5 mmol/mol, p = 0.006). SCOFF positive patients showed on average higher HbA1c levels in those models including the PHQ-9 score, but the association between SCOFF and HbA1c was not statistically significant. However, positive associations between low and middle SES and HbA1c were observed (significant only for middle SES; β_M4 men low SES_ 0.7, p = 0.146; β_M4 men middle SES_ 0.7, p = 0.036; reference: high SES).

**Table 4:** Multiple linear regression models for the association between mean HbA1c (dependent variable), PHQ-9 score and SCOFF positive screening results for eating disorders (independent variables).

	Model 1		Model 2		Model 3		Model 4		Model 5	
	ß (95% CI)	p	ß (95% CI)	p	ß (95% CI)	p	ß (95% CI)	p	ß (95% CI)	p
Male Patients										
PHQ-9 score			**0.169**	**<0.001**	**0.163**	**<0.001**	**0.166**	**<0.001**	**0.141**	**0.006**
			**(0.087; 0.252)**		**(0.080; 0.245)**		**(0.082; 0.251)**		**(0.043; 0.239)**	
SCOFF positive	0.815	0.155			0.770	0.115	0.735	0.180	0.738	0.145
	(-0.315; 1.946)				(-0.191; 1.731)		(-0.248; 1.718)		(-0.260; 1.737)	
age							-0.085	0.631	0.023	0.911
							(-0.438; 0.268)		(-0.393; 0.440)	
diabetes duration							0.073	0.649	0.017	0.922
							(-0.244; 0.389)		(-0.332; 0.366)	
BMI									-0.019	0.715
									(-0.124; 0.085)	
insulin pump therapy									0.220	0.501
(yes vs. no)									(-0.429; 0.868)	
socioeconomic status										
(reference: high)										
low									0.702	0.146
									(-0.250; 1.653)	
middle									**0.740**	**0.036**
									**(0.049; 1.432)**	
family structure/residence: living …										
(reference: with both parents)										
with mother or father (and her/his partner)									0.063	0.881
									(-0.776; 0.903)	
alone or with partner in an apartment									-0.045	0.957
									(-1.723; 1.632)	
Female Patients										
PHQ-9 score			0.069	0.071	0.056	0.165	0.054	0.183	0.035	0.410
			(-0.006; 0.143)		(-0.024; 0.136)		(-0.026; 0.134)		(-0.049; 0.119)	
SCOFF positive	0.508	0.143			0.334	0.376	0.351	0.356	0.627	0.137
	(-0.175; 1.191)				(-0.410; 1.078)		(-0.400; 1.102)		(-0.203; 1.456)	
age							0.292	0.174	-0.366	0.120
							(-0.714; 0.131)		(-0.830; 0.097)	
diabetes duration							0.188	0.355	0.193	0.354
							(-0.213; 0.589)		(-0.219; 0.605)	
BMI									-0.060	0.289
									(-0.172; 0.052)	
insulin pump therapy									0.004	0.989
(yes vs. no)									(-0.656; 0.665)	
socioeconomic status										
(reference: high)										
low									**1.228**	**0.010**
									**(0.300; 2.156)**	
middle									0.410	0.340
									(-0.438; 1.259)	
family structure/ residence: living …										
(reference: with both parents)										
with mother or father (and her/his partner)									-0.524	0.214
									(-1.355; 0.308)	
alone or with partner in an apartment									-0.152	0.760
									(-1.132; 0.829)	
other									-0.210	0.822
									(-2.059; 1.638)	

Data are presented as regression coefficient β (95% CI) with p-value; separate models for PHQ-9 score and MDS/ODS as independent variables. Model 1: SCOFF (ED) (unadjusted). Model 2: PHQ-9 score (unadjusted). Model 3: SCOFF + PHQ-9 score (unadjusted). Model 4: Model 3 + age + diabetes duration. Model 5: Model 4 + BMI + insulin pump therapy + socioeconomic status + family structure/residence

Among female patients, neither depressive symptoms nor ED symptoms were significantly associated with metabolic control. Again, HbA1c was increased in women with low and middle SES compared with those with high SES (significant only for low SES; β_M4 women low SES_ 1.2, p = 0.010; β_M4 women middle SES_ 0.4, p = 0.340).

## Discussion

Focusing on young adults with early-onset T1D, this study shows the frequent co-occurrence of depressive and ED symptoms and their varying degrees of association with metabolic control. The inclusion of female and male participants enabled the first comparative analyses to provide important gender-specific insight into screening and the advancement of therapies.

### Screening prevalences of eating problems and depression (hypothesis 1)

Despite a higher mean age of four years, the screening prevalence of EDs was only slightly lower in the recent sample than in the previously studied cohort of adolescents (age 11–17 years) with concordant inclusion criteria (women/girls: 30.2% vs. 31.2%, men/boys: 9.4% vs. 11.7%) [23]. Notably, the prevalence of depressive symptoms also appears to be comparable between the young women in this study and a previously analyzed group of adolescent girls (13.5% vs. 12.2%), despite the average disease onset in the referred study being three years later and the average disease duration being five years briefer [1]. Thus far, it remains unclear (1) whether the early manifestation and long duration of diabetes have a preventive effect on the development of psychiatric disorders in early adulthood and (2) how mental health progresses during subsequent development. Although the previously observed higher screening prevalence of EDs in women [24,25] was confirmed in this study, the number of positive screenings for depressive symptoms did not significantly differ between genders while severity of depressive symptoms was slightly higher among women. The gender difference in the predisposition for eating problems may be attributable to differences in body dissatisfaction, self-esteem or BMI that tend to be observed in the general population [24].

### Association between ED and depressive symptoms (hypothesis 2)

The association between the ED and depressive symptoms observed in the current study is in line with the results for adolescent girls reported by Colton et al. (prevalence of depressive symptoms among girls with vs. without DEB: 69.2% vs. 22.0% compared with 27.8% and 8.2% of the young adult women with and without ED symptoms in the present study) [1]. After adjusting for age, diabetes duration, BMI, SES and family structure/residence, depression severity (PHQ-9 score) was significantly associated with SCOFF among women but not among men, thus indicating gender differences in the pathophysiology of both diseases. However, the results should be interpreted with caution because of the small sample size.

### Associations between positive screening results and metabolic control (hypothesis 3)

Despite the higher screening prevalence of EDs and the significant associations between ED and depressive symptoms in women with early-onset T1D compared with those observed in the male group, the associations between the PHQ-9 score and HbA1c were significant only among men. In this group, a one-unit improvement in depression severity was associated with an HbA1c decrease up to 0.71% (corresponding to 7.7 mmol/mol), thus reducing the risk for late diabetes-related complications. As noted previously, the results of previous studies on the association between depression and metabolic control have been contradictory, and differences in the study population and the study design limit the comparability of the results [7]. In addition, the association between the symptoms of EDs, depression and metabolic control has not yet been analyzed among intensely treated male adolescents or young adults. Substantiating these results will thus be the task of future research.

One explanation for the apparent contradiction between the screening prevalence of EDs and depression severity on the one hand and metabolic outcomes on the other hand may pertain to gender differences in the expression of feelings [2]. The stronger association between ED and depressive symptoms and metabolic control observed among men compared with women may thus be attributable to gender differences in the reporting and evaluation of the severity of depressive symptoms.

### Strengths and limitations

One strength of our study was the nationwide assessment of a well-defined sample of young adults. Furthermore, although the participants were homogenous in terms of their diabetes onset and current age, they received different types of diabetes therapy at various treatment facilities throughout Germany, thus increasing the generalizability of the study results. Additional strengths are the detailed analysis of depressive symptoms and the gender-specific presentation of the results.

However, this cross-sectional study design did not allow for inferences on causality. In a prospective study on the persistence of DEB in mid-adolescent students from the general population, baseline depressiveness predicted the onset and persistence of DEB among 19-year-old women [25]. However, in another study, a loss of control over eating in children (mean age 10.4 years) was associated with depressive symptoms 4.7 years later [54].

Additional limitations of the present study include the pending complete validation of the SCOFF questionnaire for people with diabetes. Furthermore, the question regarding the choice of cut-off values used for generic screening instruments in diabetes care remains open to debate. To maintain comparability with healthy people and people with other chronic diseases, the original cutoff values for the SCOFF questionnaire and the PHQ-9 were maintained. However, because the answer to one SCOFF question (“Would you say that food dominates your life?”) may be biased owing to the demands of diabetes therapy, the cutoff value commonly used for the SCOFF questionnaire may be too low for patients with diabetes, indicating DEB rather than overt EDs [54].

Because of the nationwide sample and the restriction to questionnaires, it was not possible to evaluate positive screening results with additional diagnostic procedures. Therefore, some patients with positive screening results may not actually suffer from an ED. On the contrary, insulin restriction, as diabetes-specific purging behavior, has not been considered when estimating the prevalences of EDs; however, detailed results regarding this behavior have been previously published [23].

Another limitation of the study is the considerable non-response rate, which limits the generalizability of the study results, and the limited available information regarding non-participants. Assuming that people with symptoms of EDs, depression and/or poor metabolic control disproportionally often refuse participation in the comprehensive questionnaire study, the screening prevalences of EDs and depression and their association to metabolic control may have been underestimated.

### Implications

Aged 18 to 21, the participants in this study fell within the age range of the typical transition from pediatric to adult diabetes care. The study results indicate the relevance of a structured transition process that addresses medical, psychosocial and psychological aspects, although the participants’ early disease onset and long diabetes duration may have resulted in better disease adaptation. On the other hand, patients with early-onset T1D and long disease duration may be predisposed to diabetes distress or burnout, especially during the transition to adulthood. Regular screening for mental health problems and psychological support during the challenging period of emerging adulthood may contribute to the prevention or at least the early detection of ED or depressive symptoms [7]. Targeted education programs for young adults with T1D may support them in assuming full individual responsibility for the management of their disease, thus improving diabetes self-management and avoiding frustrating metabolic outcomes.

In conclusion, among young adults with early-onset, long-duration type 1 diabetes, ED and depressive symptoms were found to be interrelated and these symptoms were also associated with worse metabolic control, particularly among men. To prevent the persistence of these mental health problems, poorer diabetes outcomes and the early onset of diabetes complications, continuous diabetes care that includes regular screening for mental health problems during the transition to adult diabetes care is recommended.
